# An Ecological Momentary Intervention for Smoking Cessation: Evaluation of Feasibility and Effectiveness

**DOI:** 10.2196/jmir.6058

**Published:** 2016-12-12

**Authors:** Michael S Businelle, Ping Ma, Darla E Kendzor, Summer G Frank, Damon J Vidrine, David W Wetter

**Affiliations:** ^1^ Department of Family and Preventive Medicine University of Oklahoma Health Sciences Center Oklahoma City, OK United States; ^2^ Oklahoma Tobacco Research Center Stephenson Cancer Center Okahoma City, OK United States; ^3^ Division of Population Health Children's Medical Center Dallas, TX United States; ^4^ Department of Population Health Sciences and the Huntsman Cancer Institute University of Utah Salt Lake City, UT United States

**Keywords:** smartphone, mobile applications, smoking cessation, low income population

## Abstract

**Background:**

Despite substantial public health progress in reducing the prevalence of smoking in the United States overall, smoking among socioeconomically disadvantaged adults remains high.

**Objective:**

To determine the feasibility and preliminary effectiveness of a novel smartphone-based smoking cessation app designed for socioeconomically disadvantaged smokers.

**Methods:**

Participants were recruited from a safety-net hospital smoking cessation clinic in Dallas, Texas, and were followed for 13 weeks. All participants received standard smoking cessation clinic care (ie, group counseling and cessation pharmacotherapy) and a smartphone with a novel smoking cessation app (ie, Smart-T). The Smart-T app prompted 5 daily ecological momentary assessments (EMAs) for 3 weeks (ie, 1 week before cessation and 2 weeks after cessation). During the precessation period, EMAs were followed by messages that focused on planning and preparing for the quit attempt. During the postcessation period, participant responses to EMAs drove an algorithm that tailored messages to the current level of smoking lapse risk and currently present lapse triggers (eg, urge to smoke, stress). Smart-T offered additional intervention features on demand (eg, one-click access to the tobacco cessation quitline; “Quit Tips” on coping with urges to smoke, mood, and stress).

**Results:**

Participants (N=59) were 52.0 (SD 7.0) years old, 54% (32/59) female, and 53% (31/59) African American, and 70% (40/57) had annual household income less than US $16,000. Participants smoked 20.3 (SD 11.6) cigarettes per day and had been smoking for 31.6 (SD 10.9) years. Twelve weeks after the scheduled quit date, 20% (12/59) of all participants were biochemically confirmed abstinent. Participants responded to 87% of all prompted EMAs and received approximately 102 treatment messages over the 3-week EMA period. Most participants (83%, 49/59) used the on-demand app features. Individuals with greater nicotine dependence and minority race used the Quit Tips feature more than their counterparts. Greater use of the Quit Tips feature was linked to nonabstinence at the 2 (*P*=.02), 4 (*P*<.01), and 12 (*P*=.03) week follow-up visits. Most participants reported that they actually used or implemented the tailored app-generated messages and suggestions (83%, 49/59); the app-generated messages were helpful (97%, 57/59); they would like to use the app in the future if they were to lapse (97%, 57/59); and they would like to refer friends who smoke to use the Smart-T app (85%, 50/59). A minority of participants (15%, 9/59) reported that the number of daily assessments (ie, 5) was “too high.”

**Conclusions:**

This novel just-in-time adaptive intervention delivered an intensive intervention (ie, 102 messages over a 3-week period), was well-liked, and was perceived as helpful and useful by socioeconomically disadvantaged adults who were seeking smoking cessation treatment. Smartphone apps may be used to increase treatment exposure and may ultimately reduce tobacco-related health disparities among socioeconomically disadvantaged adults.

## Introduction

Tobacco use is the leading cause of preventable death in the United States [1]. Although the prevalence of smoking has declined to 15.2% among US adults who are not living in poverty, 26.3% of those living in poverty are current smokers [2]. Numerous studies have shown that low socioeconomic status (SES) and financial strain are associated with a reduced likelihood of smoking cessation (ie, [3-8]) despite the fact that individuals of low SES are just as likely to make a quit attempt [9,10]. Even when smoking cessation interventions are specifically designed for low SES populations, quit rates have been low (eg, biologically confirmed 7-day point prevalence abstinence rates of 7%-13% at 6-month follow-up [11-13]). As such, innovative smoking cessation interventions are needed to help socioeconomically disadvantaged individuals quit smoking.

Smartphone-based smoking cessation apps could play a significant role in improving cessation rates for current and future generations of smokers. Approximately 779,000 individuals download smoking cessation apps onto personal smartphones each month worldwide [14], and lower SES individuals are the fastest growing group of smartphone owners in the United States [15]. In fact, smartphone ownership more than doubled to 50% between 2011 and 2015 in households earning less than US $30,000 per year [15]. Smartphone apps could offer easily accessible, highly tailored, and intensive interventions at a fraction of the cost of traditional smoking cessation counseling, thereby overcoming many of the barriers that have hampered the use of traditional empirically supported smoking cessation treatments among lower SES individuals [16,17].

Ecological momentary assessment (EMA), in which handheld devices (eg, smartphones) are used to capture moment-to-moment experience, allows for the measurement of phenomena in real time within natural settings [18,19]. EMA data may facilitate a better understanding of the mechanisms involved in successful cessation attempts, those affecting smoking lapses, and those implicated in the transition from lapse to relapse. Although multiple studies have identified momentary predictors of smoking cessation and smoking relapse (eg, [20-24]), to our knowledge, no studies have used a participant’s responses to EMAs to automatically prompt tailored smoking cessation interventions in real time. EMAs are often used to assess individuals at multiple time points throughout a day. Thus, momentary changes in key variables can be tracked and potentially used to initiate interventions as they are needed. Using smartphones to detect high relapse risk situations and automatically deliver tailored smoking cessation interventions may help socioeconomically disadvantaged smokers to quit.

The primary objectives of this study were to evaluate the feasibility and effectiveness of the Smart Treatment app (ie, Smart-T), a novel adjunctive, tailored, smartphone-based smoking cessation intervention for smokers of low SES participating in a smoking cessation program at a safety-net hospital clinic. All study participants received usual tobacco cessation clinic care and a smartphone with the Smart-T app.

## Methods

### Participants and Procedure

This was a nonrandomized feasibility study in which all participants received the Smart-T intervention. Participants were recruited (June 2014 to May 2015) from an established safety-net hospital smoking cessation clinic in Dallas, Texas. Safety-net hospitals provide health care services regardless of ability to pay and, therefore, primarily serve individuals who are uninsured or receiving Medicaid benefits. Participants were included in the study if they (1) earned a score of ≥4 on the Rapid Estimate of Adult Literacy in Medicine–Short Form (REALM-SF) instrument [25], indicating higher than sixth-grade English literacy level; (2) were willing to quit smoking 7 days after their first clinic visit; (3) were ≥18 years of age; (4) had an expired carbon monoxide (ie, CO) level of ≥8 ppm suggestive of current smoking; (5) were currently smoking ≥5 cigarettes per day; and (6) were willing and able to attend 6 weekly assessment sessions (ie, week −1, quit day, week +1, week +2, week +3, week +4) and the 12-week follow-up session. Participants received US $30 gift cards for completing the week −1, quit day, week +4, and week +12 assessment visits. This study was approved by the institutional review boards at the University of Texas Southwestern Medical Center and the University of Texas School of Public Health.

Individuals attending the orientation visit of the Parkland Smoking Cessation Clinic were provided with detailed information about the study and given the opportunity to have their questions answered in a private room to ensure confidentiality. Written informed consent was obtained. All participants were provided with a smartphone (Samsung, Galaxy Light) for 3 weeks (ie, 1 week before cessation and 2 weeks after cessation). Participants were asked to complete 5 smartphone-prompted EMAs per day (see description of EMA types and items below). All participants were given instructions on how to use the study phone and Smart-T app features at the baseline, quit date, and 1-week follow-up visits. Specifically, participants watched a brief video on a tablet computer, created by the research team, that demonstrated general use of the smartphone and how to access and use Smart-T features. Participants also completed practice EMAs and received hands-on guidance on accessing Quit Tips and other smartphone features (see below). Finally, a link to a brief video tutorial appeared on the home screen of each smartphone so that participants could access smartphone instructions at any time. Participants could use the smartphone to make and receive calls, text, and access the Internet all free of charge. Participants were compensated upon the return of the smartphone based on the percentage of prompted EMAs that were completed over the 3-week EMA period. Specifically, those who completed 50%-74% of assessments received a US $40 gift card, those who completed 75%-89% of assessments received a US $80 gift card, and those who completed 90% or more of their assessments received a US $120 gift card. Participants were not compensated for completing participant-initiated assessments (ie, urge assessments, prequit smoking assessments, lapse assessments) or accessing on-demand features (eg, Quit Tips, Phone a Counselor).

### Description of Standard Care

The Parkland Hospital smoking cessation program offers all components of an intensive tobacco treatment recommended by the Clinical Practice Guideline [26], including (1) initial assessment of willingness to participate, (2) the use of multiple types of clinicians (eg, medical, nonmedical), (3) at least four counseling sessions, in an individual or group format, that are greater than 10 minutes in duration, (4) counseling that includes problem-solving, skills training, and social support components, and (5) the opportunity to use effective medications to aid in tobacco cessation (eg, nicotine patch, varenicline). Specifically, smokers were referred (usually by their treatment providers) to the tobacco cessation program. Participants attended one initial orientation and educational session provided by a respiratory therapist, followed by weekly group support sessions facilitated by social workers. Participants were seen individually by a physician (or other prescribing provider) on a regular basis to discuss/prescribe medication and to follow up on participant progress with smoking cessation.

### Description of Smart-T App Features

The Smart-T app contains multiple components including an EMA delivery and data transfer system, automated messages, and on-demand content. Each of these features is described below.

#### Ecological Momentary Assessment

Three types of assessments were used in this study: daily diary, random sampling, and event sampling (ie, precessation smoking, urge, postcessation lapse). Daily diary and random assessments were initiated by the Smart-T app. Specifically, the phone audibly and visually cued each daily diary and random assessment for 60 seconds. If the participant did not respond, the assessment was recorded as missed. Daily diary assessments were completed once every day, 30 minutes after waking. Random assessments were initiated 4 times per day during each participant’s normal waking hours. Event sampling assessments were initiated by participants. During the first week of assessment (prequit week), participants were instructed to indicate when they were about to smoke (by clicking the “Record Cigarette” button) immediately before smoking each cigarette. Because the assessment burden would be excessive for heavy smokers if each smoking occasion were assessed, the smartphone randomly sampled up to 2 smoking occasions from each participant per day. After completing the precigarette assessment for selected cigarettes, smokers were instructed to smoke as usual. The phone automatically prompted the postcigarette assessment 15 minutes after the precigarette assessment was completed. During the postquit period, participants were instructed to click the “Urge” button when they had an urge to smoke or they felt like they almost smoked and the “About to Slip” or “Already Slipped” buttons when appropriate during the postquit period. “About to Slip” assessments were followed by a second assessment 15 minutes later to assess whether participants actually smoked. All assessments (excluding daily diary assessments) were expected to require approximately 2-3 minutes to complete. Daily diary assessments were expected to require approximately 5 minutes to complete. All assessments were date and time stamped for future analyses. Assessment items were selected based on their hypothesized relations to smoking behavior and temptation and lapse episodes. The items assessed smoking urges, affect, stress, cigarette availability, recent alcohol use, cessation motivation, and related constructs. This EMA methodology is similar to that developed by Shiffman et al [27,28] and Stone et al [29] and has been used by our research team in many previous studies [21,22,30-34].

#### Automated Messages

For this study, 4 levels of automated messages were developed, and 1 message was delivered at the end of each EMA. Level 0 messages were pushed during the 1 week prequit period *and* during the postquit period when a participant indicated that he or she lapsed and was no longer interested in quitting smoking. During the prequit week, these messages were not tailored but rather were delivered in a predetermined order. Message topics were primarily motivational in nature and focused on planning and preparing for the quit attempt and benefits of quitting (eg, “It’s OK to have mixed feelings about quitting. Don’t let that stop you! There will be times that you don’t feel like quitting! Stick with it anyway!”).

During the 2-week postquit period, participants received individually tailored automated messages based on their EMA responses (see [35] for a complete description of the lapse risk estimator). Level 1 messages were delivered when EMA responses indicated a low level of imminent smoking lapse risk, and message content focused on maintaining abstinence motivation and general cessation advice (eg, seeking social support for cessation, coping with various lapse triggers, and benefits of quitting). Level 2 messages were delivered when EMA responses indicated a high imminent risk of smoking lapse *or* the participant already smoked that day or the day before *or* the participant indicated on the daily diary assessment that he or she had a greater than 25% chance of smoking that day. Level 2 messages were also delivered at the end of participant-initiated Urge EMAs, About to Slip EMAs, and Already Slipped EMAs. Level 2 messages primarily focused on ways to cope with current lapse triggers (ie, reported during the current EMA) and were tailored to the highest rated of 4 current lapse triggers (ie, elevated negative affect/stress, elevated smoking urge, easy access to cigarettes, or low motivation to quit). In instances where multiple triggers were equally highly rated, 1 message was delivered with preference given to negative affect/stress, smoking urge, cigarette availability, and motivation to quit, in the given order. Level 3 messages were delivered after lapse occurred and these motivational messages encouraged a return to abstinence (eg, “A slip is a sign that you need to improve your smoking cessation plan. Think about what went wrong and develop a stronger plan to stay quit. Keep trying and YOU WILL SUCCEED!”).

#### On-Demand Content

**Figure 1:**
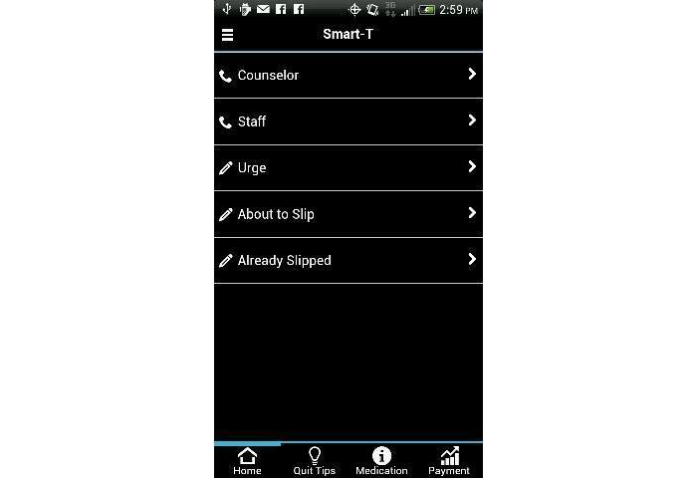
Smart-T postquit home screen.

**Figure 2:**
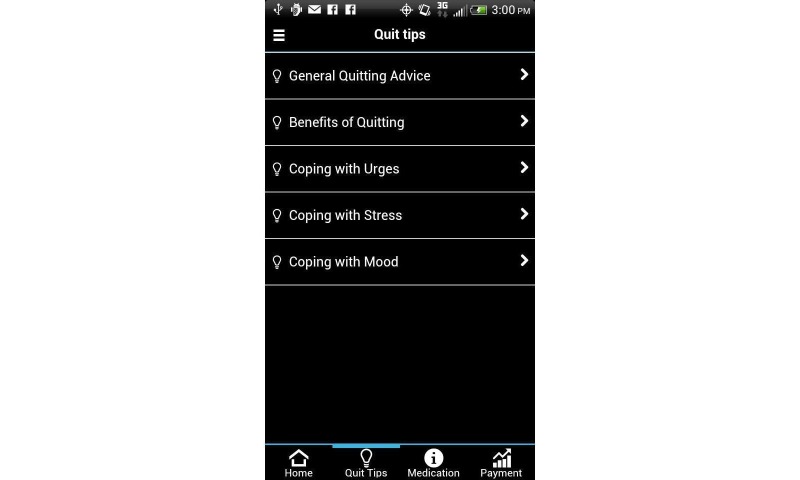
Quit Tips function.

**Figure 3:**
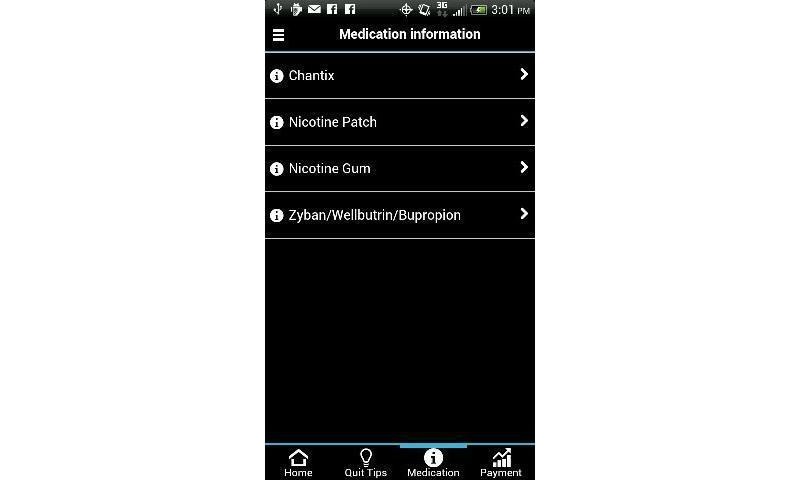
Medication function.

Several Smart-T components were available through the study-provided smartphone 24 hours a day, 7 days a week (see Figure 1). First, the “Phone a Counselor” function/button was programmed to automatically call the free Texas Tobacco Quitline (1-877-Yes-Quit) so that participants could reach a live counselor at any time. Second, the “Quit Tips” function/button accessed a menu of treatment-related messages that focused on general smoking cessation advice, various benefits of quitting, and specific suggestions on how participants might cope with stress, urges, and negative mood (see Figure 2). Third, a “Medications” function/button offered information (eg, common side effects, quit statistics, use instructions) about smoking cessation medications that were regularly prescribed by the Parkland Hospital Smoking Cessation Clinic (see Figure 3).

#### Other App Features

When pressed, the “Call Staff” function/button (see Figure 1) automatically called study staff. Participants were instructed to use this function when they had problems with the phone or the Smart-T app. Pressing the “Payment” button opened a window that indicated the number of EMAs that were prompted and completed and the current level of compensation based on the up-to-the-moment percentage of EMAs completed. In addition, during the prequit period, participants received a daily message that shared the number of days until the participant’s quit date (eg, “You are scheduled to quit smoking in 5 DAYS at 10:00 pm next Sunday night. Developing a plan to quit and taking your medications will GREATLY increase your chances for staying quit.”).

### Measures

All participants answered demographic questions at the baseline visit including age, sex, race, income, employment status, history of homelessness, and current housing status. In addition, participants completed the Heaviness of Smoking Index (HSI) and the Center for Epidemiological Studies Depression (CES-D) scale at the baseline visit. The HSI is a 2-item measure that is commonly used to assess nicotine dependence [36]. Scores range from 0 to 6 and scores ≥4 indicate moderate to high dependence. The CES-D Short Form is a 10-item measure that is commonly used to assess depressive symptoms [37]. Scores range from 0 to 30 and scores ≥10 indicate clinically significant depression.

At the 2-week follow-up visit, participants were asked a number of questions to gauge their level of satisfaction with and receptiveness to the Smart-T app and app features. Specifically, participants were asked the following: (1) How often they used the automated suggestions that followed each EMA (6-point scale from “Never” to “Always”); (2) Whether the number of EMAs was “Too high,” “About right,” or “Not enough”; (3) If the EMAs made them more aware of their thoughts, feelings, and behaviors (4-point scale from “Definitely no” to “Definitely yes”); (4) Whether the app helped them to make decisions that were supportive of quitting and staying quit (4-point scale from “Definitely no” to “Definitely yes”); (5) Whether they thought the app was “annoying” (5-point scale from “Not at all” to “Extremely”); (6) If they would recommend the app to a friend (7-point scale ranging from “Extremely unlikely” to “Extremely likely”); and (7) Whether they would be interested in using the app in the future if needed (5-point scale from “Not at all interested” to “Extremely interested”). Participants were also asked if they used each of the on-demand app features. Those who reported using particular features were asked about the usefulness of the feature. Answer options ranged from “Not at all useful” to “Extremely useful” on a 5-point scale. Finally, participants were asked, “At the end of every assessment, the phone automatically offered a tip or suggestion about smoking or smoking cessation. Overall, how helpful were these messages?” Answer options ranged from “Not at all helpful” to “Extremely helpful” on a 5-point scale.

On the quit date, participants were asked if they smoked “even a puff” since 10:00 pm on the night before their quit date visit. Participants were asked if they smoked “even a puff” during the past 7 days at each visit following the scheduled quit date (ie, postquit weeks 1, 2, 3, 4, and 12). Expired breath was tested for CO at each visit using a Vitalograph CO monitor (Lenexa, KS). Self-reported abstinence over the specified time period and a CO reading below 8 ppm (10 ppm on the quit date) were required to be considered abstinent.

### Statistical Analyses

Demographic variables, on-demand app feature usage, and participant perceptions of the app are summarized using the mean (SD) and the median for continuous variables and frequency (%) for categorical variables. The frequency with which each participant used each of the app features (ie, Quit Tips, Medication Tips, Phone a Counselor) was calculated, as was the average number of tips seen by each participant during each tip viewing session (ie, viewing intensity). Similar to many count measures, on-demand app use (eg, number of times each tip category was accessed, number of tips seen) was not normally distributed; therefore, negative binomial regression was used to identify differences in app use. These analyses were used to determine if on-demand app use (ie, frequency and intensity of overall Quit Tip and Medication Tip use) was associated with demographic variables and CO confirmed smoking status on the quit date and weeks 2, 4, and 12 postquit visits. When outliers were observed in modeling associations with on-demand feature use, the analysis was repeated after excluding outliers. Data were analyzed using SAS (version 9.4, SAS Institute Inc).

## Results

### Participants

A total of 61 participants were enrolled in this study. Of these, 2 participants (a 33-year-old African American female and a 54-year-old white male) did not complete any EMAs and were thus excluded from study analyses (sample N=59). On average, participants were 52.0 (SD 7.0) years old, African American (53%, 31/59), and female (54%, 32/59). Most participants were unemployed (78%, 46/59), 70% (40/57) earned less than US $16,000 per year, and half (49%, 29/59) had a history of homelessness. Nearly half (44%, 26/59) of the participants had significant symptoms of depression as measured by the CES-D. Nearly all study participants possessed activated cell phones (93%, 55/59) and 71% (42/59) of all participants possessed activated smartphones. At baseline, participants smoked 20.3 (SD 11.6) cigarettes per day, had been smoking for 31.6 (SD 10.9) years, had low to moderate nicotine dependence (HSI mean 3.5, SD 1.4), and had high levels of expired carbon monoxide (mean 18.6 ppm, SD 13.0).

### Attrition and Ecological Momentary Assessment Completion Rates

Most participants attended the quit date (92%, 54/59), week 1 (98%, 58/59), week 2 (98%, 58/59), week 4 (83%, 49/59), and week 12 (78%, 46/59) follow-up visits. Overall, participants were very responsive to prompted EMAs (87% of all prompted EMAs were completed) and EMA completion rates were not related to in-person treatment attendance (*P*=.17). All study phones were returned. Daily diary EMAs were completed in 6.1 (SD 1.9) and 4.8 (SD 1.5) minutes for pre- and postquit assessments, respectively. Random EMAs were completed in 2.3 (SD 0.6) and 2.1 (SD 0.9) minutes for pre- and postquit assessments, respectively. Interestingly, completion rates for the longer daily diary assessments (92% completed) were slightly higher than the shorter random assessments (85% completed). On average, participants self-initiated 15.5 assessments (ie, cigarette, urge, and lapse assessments) and completion times varied by type of participant-initiated EMA. The longest participant-initiated EMA type (ie, Already Slipped assessment) was completed in 4.5 (SD 1.5) minutes on average, while the shortest (ie, Urge assessment) was completed in 2.0 (SD 0.9) minutes. As indicated by *t* tests, there was no relation between cell phone ownership (yes vs no) and EMA completion rates (*P*=.24). However, EMA completion rates differed between smartphone owners and nonowners (*P*=.04), such that smartphone owners completed 10% more assessments than nonowners.

### Smart-T Feature Utilization

#### On-Demand Feature Use

Participants with higher levels of nicotine dependence (ie, higher scores on the HSI) accessed the Quit Tips feature more frequently (6.32 vs 2.13) than those with lower nicotine dependence (*P*<.01). In addition, nonwhite participants accessed the Quit Tips feature twice as often (nonwhite mean 6.53 times vs white mean 3.05 times; *P*=.05) and accessed the Medication Tips nearly 3 times more frequently (nonwhite mean 5.56 vs white mean 1.95; *P*<.01) than white participants. No other demographic variables (eg, income, education) were related to frequency or intensity of Quit Tip or Medication Tip use.

Most participants (83%, 49/59) accessed the Quit Tips feature at least once during the 3-week smartphone use period. Quit Tips were accessed an average of 6.5 (SD 8.0) times per participant (median 4), and an average of 31 (SD 54.1) tips were viewed on each occasion (median 18). When stratified by Quit Tip type, “Coping with Urges” was accessed by the greatest number of participants (n=36), while the highest number of tips viewed per participant per occasion was the General Quitting Advice tip type. The number of times each feature was accessed and number of tips viewed per occasion are reported in Table 1. Means and medians are reported because of outliers who sometimes viewed very large numbers of tips. Most participants (83%, 49/59) accessed the Medication Tips feature on at least one occasion. This feature was accessed 5.2 (SD 5.3) times on average (median 4) and participants viewed 15.4 (SD 31.1) tips per occasion (median 9.8). Interestingly, many participants viewed Medication Tips for medications that were not prescribed for them (see Table 1). Phone log data indicated that very few participants used the “Phone a Counselor” function. Only 13 participants made at least one call to the quitline (ie, 2 minutes or longer) during the 3-week ecological momentary intervention period. A total of 18 calls were made by these 13 participants (mean 8.8 minutes per call; median 3.1 minutes per call).

**Table 1:** On-demand Quit Tip and Medication Tip use.

Tip type		Number of participants who used the feature	Participants who viewed tips but were not prescribed the medication	Number of times a feature was accessed		Number of tips viewed per occasion	
		n (%) or n	n (%)	Mean (SD)	Median	Mean (SD)	Median
**Quit Tips**		49 (83)	N/A^a^	6.5 (8.0)	4	31.1 (54.1)	17.8
	General Quitting Advice	30	N/A	2.4 (2.6)	2	45.5 (82.4)	15
	Benefits of Quitting	21	N/A	1.8 (1.2)	1	26.0 (23.4)	20
	Coping with Urges	36	N/A	2.3 (1.7)	2	22.3 (18.9)	16
	Coping with Stress	25	N/A	2.4 (2.6)	2	9.9 (7.9)	9
	Coping with Mood	31	N/A	2.1 (2.0)	2	13.9 (9.1)	11
**Medication Tips**		49 (83)		5.2 (5.3)	4	15.4 (31.1)	9.8
	Varenicline (Chantix)	33	15 (45)	2.9 (4.1)	2	12.9 (14.2)	10
	Nicotine Patch	27	9 (33)	2.9 (2.4)	2	44.4 (164.2)	11
	Nicotine Gum	16	12 (75)	1.9 (1.9)	1	4.9 (4.0)	4
	Bupropion (Zyban)	26	16 (62)	2.0 (1.5)	1.5	7.0 (8.9)	4

^a^N/A: not applicable.

#### Automated Messages

On average, participants received 102.1 (SD 23.7) automated intervention messages following EMAs during the 21-day EMA period.

### Participant Perceptions of the Smart-T App

Participants answered questions about the usability and helpfulness of the Smart-T app and particular app features 2 weeks after the scheduled quit date (after completing EMAs and using the app for 3 weeks; the phone was returned on this date). Participants reported that the app-generated messages were helpful (97%, 57/59) and encouraged decisions that were supportive of quitting and staying quit (93%, 55/59). Most participants (90%, 53/59) reported that the app made them more aware of their thoughts, feelings, and behaviors, and 83% (49/59) reported that they used the app-generated tailored messages during the postquit period “sometimes, fairly often, very often, or always.” Most participants reported that the number (ie, 5) of daily EMAs that were prompted by the app was “about right” (75%, 44/59) or “not enough” (10%, 6/59), while 15% (9/59) reported that the number of assessments was “too high.” A minority of participants (14%, 8/59) reported that the Smart-T app was “very” or “extremely” annoying. Finally, most participants reported that they would like to use the Smart-T app in the future if needed (97%, 57/59), and 85% (50/59) would recommend friends who smoke to use the Smart-T app to help them quit. Table 2 displays self-reported use of app features and participant ratings of the usefulness of each feature. Note that only those who reported using each feature were asked about the perceived utility of that feature.

**Table 2:** Self-reported app feature use and usefulness.

Function	Self-reported use of the function (N=59) n (%)	How helpful or useful was the function?		
		Answer option	n (%)	Mean (SD)
Automated Messages	59 (100)	Not at all helpful	2 (3)	3.8 (1.0)
		Slightly helpful	4 (7)	
		Moderately helpful	15 (25)	
		Very helpful	22 (37)	
		Extremely helpful	16 (27)	
Quit Tips	45 (76)	Not at all useful	0 (0)	3.6 (1.0)
		Slightly useful	9 (20)	
		Moderately useful	10 (22)	
		Very useful	16 (36)	
		Extremely useful	10 (22)	
Medication Tips	38 (64)	Not at all useful	1 (3)	3.5 (1.0)
		Slightly useful	6 (16)	
		Moderately useful	12 (32)	
		Very useful	13 (34)	
		Extremely useful	6 (16)	
Phone a Counselor	10 (17)	Not at all useful	0 (0)	3.1 (1.0)
		Slightly useful	3 (30)	
		Moderately useful	4 (40)	
		Very useful	2 (20)	
		Extremely useful	1 (10)	

### Smoking Status and On-Demand Feature Use

A total of 41% (24/59), 17% (10/59), 31% (18/59), 27% (16/59), 22% (13/59), and 20% (12/59) of participants met criteria for point prevalence abstinence at the quit date, week 1, week 2, week 3, week 4, and week 12 follow-up visits, respectively. Participants who did not attend a particular follow-up visit were considered nonabstinent. Notably, the proportion of abstinent participants was significantly higher at the 2-week follow-up compared with the 1-week follow-up (McNemar *P*=<.001).

Negative binomial regression analyses indicated that there was no significant association between the frequency or intensity of tips (ie, Quit Tips and Medication Tips) viewed during the prequit period and biochemically confirmed smoking status on the scheduled quit date (*P* values ≥.40). However, there was a significant relation between the total number of Quit Tips viewed and week 12 smoking status (*P*<.01). Specifically, participants who viewed greater numbers of Quit Tips had a greater likelihood of nonabstinence at the 12-week follow-up visit. Importantly, 2 individuals were identified as extreme outliers (ie, they viewed far greater numbers of Quit Tips compared with other participants). Analyses that excluded these outliers indicated that viewing more Quit Tips was associated with greater likelihood of nonabstinence at the week 2 (*P*=.015), 4 (*P*=.001), and 12 (*P*=.027) postquit visits. No other significant associations were found between frequency or intensity of Medication Tip or Quit Tip use and smoking status at follow-up visits (all *P* values >.09).

## Discussion

### Principal Findings

Study findings indicate that this first-of-its-kind app offers a feasible way to provide tailored smoking cessation interventions to socioeconomically disadvantaged adults who are seeking to quit smoking. Over a 3-week period, participants received an intensive level of tailored and automated intervention messages (102 messages on average) and most (97%, 57/59) rated these messages as helpful. Furthermore, nearly all participants who accessed the on-demand Quit Tips (100%) and Medication Tips (97%) features rated them as being useful. Most participants (85%, 50/59) reported that they would refer a friend to use the app, and 97% (57/59) reported that they would like to use Smart-T if they were to lapse in the future. These findings are consistent with smokers’ requests for smoking cessation apps that provide on-demand messages focused on coping with cravings, motivational messages, outcome expectancies, and facts about the effects of smoking [38,39]. The Smart-T app contains each of these features. Importantly, 20% (12/59) of all participants were biochemically confirmed abstinent 12 weeks following the scheduled quit date. This rate of confirmed abstinence is promising as it is higher than what has been commonly reported in other samples of socioeconomically disadvantaged treatment-seeking smokers [11-13,40]. Tailored just-in-time treatments may offer new ways to address disparities in smoking cessation.

Overall, study participants were highly responsive to the 5 automated daily EMAs (87% completed), a rate that is above the EMA compliance benchmark set by Stone and Shiffman [41] and on par with or better than other studies that have collected EMAs in higher SES samples [42-47]. Only 15% (9/59) of all participants reported that the number of prompted EMAs (5 per day) was “too high.” This is a meaningful finding considering that the EMAs required 2 to 6 minutes to complete and could be prompted at any time during the participant’s normal waking hours. Abroms and colleagues [48] reported that 29% of participants indicated that there were “too many” texts in their pilot test of the Text2Quit smoking cessation program. In that study, participants received up to 25 text messages per week (mean 14.5 messages per week) over the first 4 weeks of the program. There are multiple reasons Smart-T participants may have been more accepting of the high number of prompts (ie, 35 per week) compared with the Text2Quit sample. First, Smart-T participants reported that they appreciated the automatic tailored messages that were delivered at the completion of each EMA. This finding is consistent with previous work that has shown that dynamically tailored interventions are more accepted and effective than static messages [49]. Second, Text2Quit participants were asked about their perceptions of the Text2Quit messages after receiving them for 4 weeks, whereas perceptions about the Smart-T app were collected after only 3 weeks. It is possible that favorable perceptions of the Smart-T app may decline with longer periods of use.

Similar to findings from a previous study [50], participants with greater nicotine dependence accessed the Quit Tips feature more times than those who were less dependent. This finding is in contrast to Zeng et al [51] who showed that heavier smoking predicted lower app use. Furthermore, nonwhite participants accessed the Quit Tips and Medication Tips features 2 to 3 times more often than white participants. It is possible that minority smokers and individuals with higher levels of nicotine dependence may have found the app to be more informative and engaging. In fact, many of the Quit Tip messages that were used for this study were originally created for a trial that examined the clinical utility of a culturally tailored palmtop computer–delivered treatment for smoking cessation among African Americans [52].

Most participants (83%, 49/59) used the on-demand Quit Tips and Medication Tips features and these participants viewed an average of 31 (median 18) Quit Tips and 15 (median 10) Medication Tips per viewing session. Participants used these on-demand features in ways that were not anticipated. For instance, it was expected that participants would access tips more frequently and only view a few tips per occasion. There are opposing views about why participants may have accessed such a large number of tips. For example, participants may have chosen to view so many tips per occasion because many tips may not have been relevant to their current situation, and they viewed tips until they found a relevant one. Alternatively, participants may have viewed such a large number of tips because they found the information useful and engaging. High participant ratings for the on-demand tip features may add weight to the latter explanation. Furthermore, it is puzzling why so many participants viewed Medication Tips for medications that were not prescribed to them. Future research should query participants about their rationale for using specific app features.

The frequency, intensity, and types of on-demand Quit Tips that were accessed warrant discussion. More participants used the “Coping with Urges” tips compared with all other types of tips. In addition, participants accessed the “Benefits of Quitting” tips fewer times than the other Quit Tip types. Furthermore, considering each tip viewing occasion, participants chose to view fewer tips that were focused on “Coping with Stress” or “Coping with Mood” compared with other tip types. These findings may provide insights about preferences for specific types of cessation information and could be used to inform future smartphone apps.

It was unexpected that so few participants (22%) would use the “Phone a Counselor” function, which offered a free one-click connection to Texas Tobacco Quitline counselors. While it is unclear why so few participants used this feature, it is possible that access to weekly counseling sessions and on-demand Smart-T Quit Tips supported the belief that accessing the quitline was unnecessary or redundant. Future research should examine if a one-click quitline counseling feature is utilized when paired with stand-alone, automated, message-based smoking cessation apps.

Although the feasibility of the Smart-T app, as an adjunct to in-person treatment, was the primary outcome of interest for this study, exploratory analyses were conducted to examine smoking cessation in this sample. Only 41% (24/59) of the participants quit smoking on their scheduled quit date and only 17% (10/59) maintained abstinence for the entire first postquit week. Interestingly, the 7-day point prevalence abstinence rate nearly doubled to 31% by the second week after cessation. This level of “recycling” or return to abstinence following an initial lapse is unusual [8,53] and may warrant further study. It may be that the Smart-T app helped some participants to overcome early lapse and return to abstinence. Alternatively, this finding may be an artifact of the small sample size of this study. Approximately 20% of this very low SES sample of patients seeking smoking cessation were abstinent at the 12-week follow-up visit. It is notable that biochemically verified abstinence rates were higher than many other interventions that have been conducted with other low SES samples [12,13,40,54,55] and on par with many interventions that have been conducted in more advantaged samples [56,57]. Furthermore, abstinence rates for this study are on par with or better than recently published studies of higher SES smokers seeking cessation that utilized text messaging, which included repeated suggestions to use quitline counseling and nicotine replacement therapy (NRT; [48,58,59]), or a stand-alone smartphone app [60]. This feasibility study is merely a first step, and more vigorous and controlled testing of the utility of the Smart-T app is needed. An ongoing pilot randomized controlled trial will compare the Smart-T app plus NRT to (1) usual in-person smoking cessation treatment (counseling plus NRT) and (2) the National Cancer Institute QuitGuide app plus NRT.

The total number of Quit Tips viewed was related to CO confirmed abstinence status at study follow-up visits. Specifically, individuals who viewed more tips during the 3-week period when participants had access to the Smart-T app were more likely than those who viewed fewer tips to be biochemically confirmed nonabstinent at the week 2, 4, and 12 postcessation follow-up visits. Previous studies that have examined smartphone-based smoking cessation apps have indicated that greater app use is related to increased likelihood for smoking lapse [61], whereas other studies have indicated that greater app use is related to a lower likelihood of lapse [50,60,62]. There are many reasons why participants might have used, or not used, Smart-T app features. First, some abstinent participants may have infrequently used the on-demand app features because of lack of perceived need, while others may have believed that the on-demand app features were integral to their maintenance of abstinence. Second, nonabstinent participants may have lost interest in quitting and thus avoided using the on-demand features, while other nonabstinent participants may have used the on-demand features to prepare them for future quit attempts. Future studies should directly assess the drivers of on-demand feature use so that more effective procedures and features can be created and added to improve cessation apps.

### Limitations

The results of this feasibility study should be considered with study limitations. First, this study was not adequately powered to examine the relation between use of Smart-T app features and lapse. Thus, statistical analyses did not control for potential confounders (eg, age, motivation) of the relationship between level of app use and smoking cessation. In addition, all study participants received a smartphone with the Smart-T app; thus, results cannot be used to determine if the Smart-T app improved cessation outcomes beyond the usual safety-net tobacco cessation clinic care. Finally, because of the budget constraints of this feasibility study, participants were only followed up for 12 weeks after their scheduled quit attempt. A well-powered randomized controlled trial that follows up smokers for at least 6 months is needed to adequately examine the utility of the Smart-T app.

### Conclusions and Future Directions

In conclusion, this intensive novel smartphone app that tailored intervention messages based on participant-reported situations and symptoms and offered on-demand access to treatment-based messaging was well-used and well-liked in a sample of socioeconomically disadvantaged (eg, 49%, 29/59, reported at least one period of homelessness in their lifetime) smokers seeking cessation treatment at a safety-net hospital. Easily accessible, highly tailored, intensive, well-liked, and low-burden smartphone-based smoking cessation apps may offer new ways to increase treatment exposure and utilization among underserved socioeconomically disadvantaged smokers who have limited access to effective smoking cessation treatments.

## Abbreviations

CES-D: Center for Epidemiological Studies Depression
EMA: ecological momentary assessment
HSI: Heaviness of Smoking Index
NRT: nicotine replacement therapy
SES: socioeconomic status
